# Prevalence and Predictors of Metabolic Syndrome in Young Asymptomatic Gujarati Population

**DOI:** 10.1155/2015/365217

**Published:** 2015-07-30

**Authors:** Sharad R. Jain, Komal H. Shah, Himanshu N. Acharya, Kaushik Barot, Kamal H. Sharma

**Affiliations:** ^1^Department of Cardiology, U. N. Mehta Institute of Cardiology and Research Center, BJ Medical College and Civil Hospital Campus, Asarwa, Ahmedabad, Gujarat 380 016, India; ^2^Department of Research, U. N. Mehta Institute of Cardiology and Research Center, BJ Medical College and Civil Hospital Campus, Asarwa, Ahmedabad, Gujarat 380 016, India

## Abstract

*Background*. Metabolic syndrome is a cluster of risk factors leading to the development of atherosclerotic cardiovascular diseases. We aimed to evaluate the prevalence of metabolic syndrome (MS) and its predictors in young and apparently healthy Gujarati individuals. *Methods*. This population based cross-sectional study involved a total of 1500 healthy adults of 20–40 years of age. Demographic details and clinical data such as body mass index (BMI), waist circumference (WC), and blood pressure were measured along with the estimations of lipoprotein (a), total cholesterol (TC), triglyceride (TG), total lipid, LDL/HDL ratio, TC/HDL ratio, and fasting blood glucose (FBS). *Results*. Overall in young Gujarati population (20–40 years) prevalence rates of MS were 16.0% (male: 21.5%; female: 10.8%) where the metabolic abnormalities increased with advanced age as 9.56% of the young population (20–30 years) had MS, in contrast to the 24.57% in the old (31–40 years). Odds ratio analysis had indicated BMI (1.120; 95% CI: 1.077–1.163; *P* < 0.0001) as the strongest risk factor for MS closely followed by advancing age (1.100; 95% CI: 1.061–1.139; *P* < 0.0001) levels. *Conclusion*. Prevalence of metabolic syndrome in young Gujarati population reinforces the need for early life style intervention and awareness programs in this ethnic group.

## 1. Introduction

The metabolic syndrome (MS) is a cluster of metabolic abnormalities possessing complex pathogenesis. The most widely documented metabolic risk factors are atherogenic dyslipidemia, hypertension, and diabetes. Atherogenic dyslipidemia is a combination of lipoprotein abnormalities including a reduced level of high-density lipoprotein cholesterol (HDL-C) and increased level of serum triglyceride (TG), apolipoprotein B, and low-density lipoprotein cholesterol (LDL-C). These characteristic abnormalities provide evidence of a prothrombotic and a proinflammatory state of an individual. Recently it was documented that about one-third of the urban Indians are suffering from MS clearly indicating the role of urbanization and mechanization in the disease onset. The overall prevalence of MS in Indian population is 31.4% where females (48.2%) are more affected than their male counterparts (16.3%) [1, 2]. The age-wise MS prevalence had increased from 2.9% in those aged 18–30 years to 31.0% in those aged 60–69 years in Asians [3].

The metabolic factors causative of MS are highly associated with cardiovascular diseases (CVD) and diabetes. These factors often lead to a 2-fold elevation in CVD risk and a 5-fold elevation in risk of diabetes (if not already present) within 5 years, with an even higher long-term risk. In urban India, nearly 77.2% of the diabetic patients had MS which was significantly higher in women (87.71%) as compared to men (69.33%), whereas in coronary artery disease (CAD) patients, the prevalence of MS was reported to be 60.06%. However, the epidemiological assessment of MS prevalence and the individual components contributing to its development in young and apparently healthy Indians is less studied [4].

In this state of affairs, the aim of the study was to assess the prevalence of MS in young and apparently healthy Gujarati Asian Indians and look for its predictors in this ethnic group of population [5].

## 2. Material and Method

### 2.1. Study Design

This observational and randomized screening study was conducted by U. N. Mehta Institute of Cardiology and Research Center. The protocol was approved and cleared by Institutional Ethics Committee. Total 1500 individuals of both genders (729 males and 1006 females), who were apparently healthy, asymptomatic, disease-free, and ranging in age from 20 to 40 years, were included in the study. The subjects taking any medications and with abnormal stress test were excluded from the investigation. The details of demographic data, ethnicity, family history of CAD, and smoking were collected for each individual.

### 2.2. Metabolic Syndrome Risk Factors Assessment

Laboratory assessments included obtaining venous blood samples in a fasted state for the determination of lipid components (total cholesterol (TC), HDL-C, LDL-C and TG, and total lipid) and blood glucose. Serum glucose and lipids were measured by International Federation of Clinical Chemistry (IFCC) approved enzymatic methods using commercially available kit on autoanalyzer (ARCHITECH PLUS ci4100, Germany). Blood pressure was measured using a sphygmomanometer and hypertension was diagnosed if the systolic blood pressure (SBP) was higher than 140 mmHg or the diastolic blood pressure (DBP) was above 90 mmHg. Waist circumference (WC) was evaluated at midway between the iliac crest and the last rib.

### 2.3. Definition and Preferred Cutoff Values

The MS was defined according to the NCEP-ATPIII guidelines with an anthropometric modification of WC value that is specifically applicable to South Asians [5]. Patients were defined as having the MS when they met at least 3 of 5 criteria: (1) elevated WC > 90 cm in men and >80 cm in women, (2) elevated blood pressure (SBP > 130 mmHg and/or DBP > 85 mmHg), (3) reduced HDL-C < 40 mg/dL in men and <50 mg/dL in women, (4) elevated fasting glucose > 110 mg/dL or drug treatment for elevated glucose, and (5) elevated TG ≥ 150 mg/dL or drug treatment for elevated TG.

### 2.4. Statistical Analysis

Statistical analysis was performed using SPSS, Version 20.0 (Chicago, IL, USA). Qualitative data were expressed as proportions whereas the quantitative data was expressed as mean ± SD. As the data follows non-Gaussian distribution the variables were compared using Mann-Whitney* U* test. The level of significance was accepted at *P* < 0.05. Binary logistic regression test was used to assess the predictors contributing to the development of MS in this population.

## 3. Result

The demographic characteristics of the study population are presented in Table 1. Out of 1500 healthy young subjects, 771 were females (51.4%) and 729 were males (48.6%) showing the overall mean age of 29.88 (±5.97) years. According to modified NCEP-ATPIII criteria, 240 (16.0%) individuals were suffering from MS. The lipid profile of the overall population showed mean value of Lp. (a), TC, TG, HDL-C, LDL-C, VLDL, and total lipid as 28.16 ± 25.81, 171.48 ± 34.76, 103.72 ± 61.82, 43.82 ± 9.93, 106.91 ± 29.28, 20.74 ± 12.36, and 619.02 ± 84.13 mg/dL, respectively. The levels of other risk factors such as blood sugar, SBP, DBP, BMI, and WC were 80.39 ± 14.24 mg/dL, 124.09 ± 16.23 mm/Hg, 79.5 mm/Hg, 23.12 ± 4.82 (kg/m^2^), and 86.80 ± 25.16 cm correspondingly. Among study participants, 155 (10.3%) had family history of premature CAD and 123 (8.2%) had addiction.

Comparison of various risk factors according to the presence or absence of MS is shown in Table 2. The population affected with MS was mostly male (65.4% versus 45.4%) and older (33.00 ± 5.33 versus 29.29 ± 5.91 years). The levels of blood sugar (89.00 ± 20.30 versus 78.75 ± 12.00), blood pressure (SBP: 137.96 ± 16.91 versus 121.45 ± 14.69, DBP: 86.60 ± 9.18 versus 78.15 ± 9.18), TC (192.75 ± 40.00 versus 167.42 ± 32.11), TG (157.37 ± 99.30 versus 93.50 ± 45.04), LDL-C (120.49 ± 32.77 versus 104.32 ± 27.85), BMI (2598 ± 3.80 versus 22.66 ± 4.82), and WC (95.63 ± 10.92 versus 85.09 ± 26.78) were significantly (<0.0001) higher in the population having MS as compared to the population not having MS. The low HDL-C (40.80 ± 9.40) level was highly prevalent in individuals detected with MS. However, no statistical significance was obtained between the values of Lp. (a), premature CAD, and addiction of both groups.

The population being affected by MS were further categorized according to their gender and the data is presented in Table 3. Results showed that females had better risk factor profile as most of the risk factors were considerably higher in males as compared to females. The risk factors associated with male gender were elevated TG (177.20 ± 108.34), HDL-C (37.45 ± 7.04), VLDL (35.45 ± 21.70), total lipid (710.24 ± 135.24), LDL/HDL ratio (3.32 ± 1.03), TC/HDL ratio (5.33 ± 1.27), SBP (139.90 ± 16.46), and increased WC (96.73 ± 10.03) along with greater incidence of premature CAD (13.4%) and addiction (11.5%). However, the level of lipoprotein (a) (34.30 ± 27.78 versus 22.92 ± 21.56) was significantly higher in females.

The distribution of risk factors causing MS according to age is indicated in Table 4. An increase in frequency of MS was observed with increasing age (20–30 years: 9.57%; 31–40 years: 24.57%). The associated risk factors of metabolic syndrome in comparatively older age group (31–40 years) were male gender (65.8%) and increased levels of TC (198.35 ± 42.06), TG (173.56 ± 111.99), VLDL (34.69 ± 22.42), total lipid (712.44 ± 135.43), TC/HDL-C ratio (5.09 ± 1.39), and DBP (87.49 ± 8.80).

The incidence of MS in individuals having diabetes, hypertension, hypertriglyceridemia, low HDL-C, and abnormal WC was also assessed. Out of 1500 patients, included in the study, 6.3%, 36.4%, 24.86.%, 15.3%, 56.5%, 47.5%, and 10.3% of the population were detected with the abnormalities of sugar, SBP, DBP, TG, HDL-C, WC, and positive history of premature CAD, respectively. From this the prevalence of MS was the highest in individuals having diabetes (51.6%) followed by TG (48.9%). These patients were further categorized according to age and gender and the results are shown in Table 5. The strength of various risk factors in the development of MS was assessed using multivariate logistic regression analysis and the results are presented in Table 6. We have found that BMI (odds ratio: 1.120; 95% CI: 1.077 to 1.163; *P* < 0.0001) and age (odds ratio: 1.100; 95% CI: 1.061 to 1.139; *P* < 0.0001) are two prime contributors of MS in this ethnic group.

## 4. Discussion

The process of MS may start in very early stage of life which continues to progress during childhood and adolescence, reaching almost 50% in prevalence in severally obese youngster [6–10]. However, this process is subjected to variation according to the region, urbanization, lifestyle patterns, and socioeconomic and cultural factors in Asian Indian community. The prevalence of metabolic syndrome is increasing exponentially in India, in both the urban and rural areas showing the prevalence range from 11% to 41% [11]. In the current study for the first time the prevalence of MS in healthy and young Gujarati Asian Indians has been reported. We found that 16.0% of the young Gujaratis who are below the age of 40 are suffering from MS. The mean blood sugar, TG, TC, LDL-C, SBP, and DBP were significantly higher and HDL-C levels were significantly lower in this population. These findings were similar to those obtained in the studies on Chennai urban population by Kota et al. and on US adult population by Chen et al. [12, 13].

We have observed that the occurrence of MS varies according to the gender as more young males (21.50%; *n* = 157) were having MS as compared to females (10.8%; *n* = 83). The higher prevalence of diabetes, dyslipidemia, and hypertension was found to be the contributor of MS in male gender, whereas young females had fairer risk factor profile due to premenopausal estrogenic protection. We have observed that male gender itself provides strong prediction of MS development in this ethnic group as maximum odds were observed with this subset of population. However as reported by others the level of Lp. (a), a factor possessing genetic association, was highly prevalent in females as matched to their males counterparts. This pattern of lipid abnormality was documented in a recent Nigerian study [14] and Roshni et al. [15] studies also. The family history of premature CAD and addiction were also significantly higher in females which may play a role in the development of the MS especially in younger woman [16].

We found that the prevalence of MS is highly associated with advancing age in both genders. It is noteworthy that the frequency of MS increases from 9.56% in 20–30-year individuals to 24.57% in 30–40-year individuals. Hildrum et al. [17] found increase in prevalence of metabolic syndrome with increasing age that 6.7% of subjects in the age group of 20–29 years are increased to 29.3% of subjects in the age group of 30–39 years. Hildrum et al. [17] findings were almost similar to this study on Norwegian population. Total cholesterol, TG, VLDL, total lipid, and TC/HDL-C had significantly higher prevalence in comparatively older group of 31–40 years compared to age group of 20–30 years.

Though the factors of MS are multifactorial, distribution of these risk factors is subjected to ethnic and racial variations. Asian Indians have higher rates of diabetes and hence are more prone to develop MS as compared to their Caucasian counterparts [18]. Diabetes mellitus is characterized by disorders of insulin action or insulin secretion, either of which may be a predominant feature. Hyperinsulinemia appears to be a compensatory mechanism that responds to increased levels of circulating glucose and hence development of MS. People who develop diabetes usually pass through the phases of excessive adipogenesis (obesity), insulin resistance, hyperinsulinemia, pancreatic beta cells stress, and damage leading to progressive decrease of insulin secretion, impaired glucose postprandial, and fasting levels [19, 20]. The second key contributor of MS development in Gujarati Asians was TG (48.9% and odds) which has long been identified as “obesity epidemic” contributing to the rising prevalence of MS in Indians. This phenomenon refers to the fact that despite the relatively lower prevalence rates of generalized obesity, Indians tend to have a greater degree of central body obesity and increased body fat, particularly increased visceral fat, and have lower cutoffs for BMI in comparison to their Caucasian counterparts [21]. Obesity contributes significantly to the development of MS, especially in presence of increased WC, as it induces insulin resistance and exerts highly deleterious impact on metabolism [22].

Collectively, diabetes and obesity are risk predictors of both venous thrombosis and an occlusive arterial disease most likely due to existence of an atherothrombotic syndrome secondary to insulin resistance and defective fibrinolysis. Patients with MS were found to have significantly more atherosclerosis compared with the patients without MS, independent of their diabetes status [23].

## 5. Conclusion

In conclusion, prevalence of metabolic syndrome in young Gujarati Asian Indian population reinforces the need for a comprehensive noncommunicable disease prevention and control program. This study has shown the unacceptably high prevalence rate of MS in males (65.4%) compared to females (34.6%) showing an accelerating trend with ageing. Increasing awareness and early identification of these clusters of risk factors should be emphasized in designing population-wide prevention strategies for Gujarati Asian Indians, in particular.

## Figures and Tables

**Table 1:** Demographic data of the study population.

Study variable	Mean ± SD, *N* (%)
Gender	
Male	729 (48.6%)
Female	771 (51.4%)
Age (year)	29.88 ± 5.97
Lp. (a) (mg/dL)	28.16 ± 25.81
Blood sugar (mg/dL)	80.39 ± 14.24
TC (mg/dL)	171.48 ± 34.76
TG (mg/dL)	103.72 ± 61.82
HDL-C (mg/dL)	43.82 ± 9.93
LDL-C (mg/dL)	106.91 ± 29.28
VLDL (mg/dL)	20.74 ± 12.36
Total lipid (mg/dL)	619.02 ± 84.13
LDL-C/HDL-C	2.59 ± 1.13
TC/HDL-C	4.12 ± 1.52
Premature CAD	155 (10.3%)
Addiction	123 (8.2%)
SBP (mm/hg)	124.09 ± 16.23
DBP (mm/hg)	79.50 ± 9.79
BMI (kg/m^2^)	23.12 ± 4.82
WC (cm)	86.80 ± 25.16
Metabolic syndrome	240 (16.0%)

Lp. (a): lipoprotein; TC: total cholesterol; TG: triglyceride; HDL-C: high-density lipoprotein cholesterol; LDL-C: low-density lipoprotein cholesterol; VLDL: very low-density lipoprotein; SBP: systolic blood pressure; DBP: diastolic blood pressure; BMI: body mass index; WC: waist circumference.

**Table 2:** Profile comparison of population with and without metabolic syndrome.

Variable	With MS, *N* = 240	Without MS, *N* = 1260	*P* value
	Mean ± SD, *N* (%)	Mean ± SD, *N* (%)	
Gender			
Male	157 (65.4%)	572 (45.4%)	<0.0001
Female	83 (34.6%)	688 (54.6%)	<0.0001
Age (year)	33.00 ± 5.33	29.29 ± 5.91	<0.0001
Lp. (a) (mg/dL)	26.86 ± 24.45	28.40 ± 26.83	0.3970
Blood sugar (mg/dL)	89.00 ± 20.30	78.75 ± 12.10	<0.0001
TC (mg/dL)	192.75 ± 40.00	167.42 ± 32.11	<0.0001
TG (mg/dL)	157.37 ± 99.30	93.50 ± 45.06	<0.0001
HDL-C (mg/dL)	40.80 ± 9.40	44.39 ± 9.92	<0.0001
LDL-C (mg/dL)	120.49 ± 32.77	104.32 ± 27.85	<0.0001
VLDL (mg/dL)	31.46 ± 19.88	18.71 ± 09.00	<0.0001
Total lipid (mg/dL)	690.92 ± 123.13	605.32 ± 66.16	<0.0001
LDL-C/HDL-C	3.10 ± 1.05	2.50 ± 1.17	<0.0001
TC/HDL-C	4.93 ± 1.37	3.96 ± 1.49	<0.0001
Premature CAD	32 (13.3%)	123 (9.8%)	0.1211
Addiction	25 (10.4%)	98 (7.8%)	0.2160
SBP (mm/hg)	137.96 ± 16.91	121.45 ± 14.69	<0.0001
DBP (mm/hg)	86.60 ± 9.81	78.15 ± 9.18	<0.0001
BMI (kg/m^2^)	25.98 ± 3.80	22.66 ± 4.74	<0.0001
WC (cm)	95.63 ± 10.92	85.09 ± 26.78	<0.0001

MS: metabolic syndrome; Lp. (a): lipoprotein; TC: total cholesterol; TG: triglyceride; HDL-C: high-density lipoprotein cholesterol; LDL-C: low-density lipoprotein cholesterol; VLDL: very low-density lipoprotein; SBP: systolic blood pressure; DBP: diastolic blood pressure; BMI: body mass index; WC: waist circumference.

**Table 3:** Influence of gender on prevalence and profile of metabolic syndrome.

Variable	Males having MS	Females having MS	*P* value
	*N* = 157	*N* = 83	
Age (year)	32.78 ± 5.27	33.40 ± 5.45	0.3925
Lp. (a) (mg/dL)	22.92 ± 21.56	34.30 ± 27.78	0.0005
Blood sugar (mg/dL)	91.10 ± 20.27	85.02 ± 19.89	0.0271
TC (mg/dL)	195.59 ± 42.56	187.40 ± 34.54	0.1325
TG (mg/dL)	177.20 ± 108.34	119.84 ± 65.06	<0.0001
HDL-C (mg/dL)	37.45 ± 7.04	47.13 ± 10.07	<0.0001
LDL-C (mg/dL)	122.69 ± 34.43	116.34 ± 29.10	0.1537
VLDL (mg/dL)	35.45 ± 21.70	23.93 ± 12.96	<0.0001
Total lipid (mg/dL)	710.24 ± 135.24	654.37 ± 85.57	0.0007
LDL-C/HDL-C	3.32 ± 1.39	2.66 ± 0.95	<0.0001
TC/HDL-C	5.33 ± 1.27	4.17 ± 1.23	<0.0001
Premature CAD	21 (13.4%)	11 (13.3%)	0.011
Addiction	18 (11.5%)	7 (8.4%)	0.032
SBP (mm/hg)	139.9 ± 16.46	134.29 ± 17.26	0.0142
DBP (mm/hg)	86.92 ± 10.69	85.99 ± 7.94	0.4864
BMI (kg/m^2^)	26.15 ± 3.52	25.66 ± 4.28	0.3429
WC (cm)	96.73 ± 10.03	91.83 ± 11.84	0.0009

MS: metabolic syndrome; Lp. (a): lipoprotein; TC: total cholesterol; TG: triglyceride; HDL-C: high-density lipoprotein cholesterol; LDL-C: low-density lipoprotein cholesterol; VLDL: very low-density lipoprotein; SBP: systolic blood pressure; DBP: diastolic blood pressure; BMI: body mass index; WC: waist circumference.

**Table 4:** Influence of age on prevalence and profile of metabolic syndrome.

Variable	Age group (20–30)	Age group (31–40)	*P* value
	*N* = 82	*N* = 158	
Gender			
Male	53 (64.6%)	104 (65.8%)	0.0026
Female	29 (35.4%)	54 (34.2%)	0.0026
Lp. (a) (mg/dL)	26.89 ± 22.99	26.84 ± 25.24	0.9880
Blood sugar (mg/dL)	83.04 ± 13.16	92.09 ± 22.59	0.0010
TC (mg/dL)	181.96 ± 33.66	198.35 ± 42.06	0.0025
TG (mg/dL)	126.17 ± 57.34	173.56 ± 111.99	0.0004
HDL-C (mg/dL)	41.33 ± 10.04	40.53 ± 9.07	0.5329
LDL-C (mg/dL)	115.39 ± 28.01	123.14 ± 34.77	0.0822
VLDL (mg/dL)	25.24 ± 11.49	34.69 ± 22.42	0.0004
Total lipid (mg/dL)	649.46 ± 80.85	712.44 ± 135.43	0.0001
LDL-C/HDL-C	2.95 ± 1.06	3.17 ± 1.04	0.1239
TC/HDL-C	4.62 ± 1.29	5.09 ± 1.39	0.0116
Premature CAD	7 (8.5%)	25 (15.5%)	0.0884
Addiction	9 (11.0%)	16 (10.1%)	0.0012
SBP (mm/hg)	137.18 ± 15.57	138.37 ± 17.61	0.6063
DBP (mm/hg)	84.88 ± 11.38	87.49 ± 8.80	0.0505
BMI (kg/m^2^)	25.93 ± 4.15	26.01 ± 3.62	0.8775
WC (cm)	94.28 ± 9.27	95.42 ± 11.69	0.4441

Lp. (a): lipoprotein; TC: total cholesterol; TG: triglyceride; HDL-C, high-density lipoprotein cholesterol; LDL-C: low-density lipoprotein cholesterol; VLDL: very low-density lipoprotein; SBP: systolic blood pressure; DBP: diastolic blood pressure; BMI: body mass index; WC: waist circumference.

**Table 5:** Prevalence of metabolic syndrome in population having diabetes, blood pressure, dyslipidemia, and abnormal waist circumference.

Variable	Total affected population	MS present	MS present in males & females	*P * value	MS present in different age groups	*P* value
DM (mg/dL)	95 (6.3%)	49 (51.6%)	M = 38 (77.6%)	<0.0001	20–30 = 5 (10.2%)	<0.0001
			F = 11 (22.4%)		31–40 = 44 (89.8%)	

SBP (mm/hg)	546 (36.4%)	188 (34.3%)	M = 128 (68.1%)	<0.0001	20–30 = 67 (35.6%)	<0.0001
			F = 60 (31.9%)		31–40 = 121 (64.4%)	

DBP (mm/hg)	373 (24.86%)	146 (39.5%)	M = 97 (66.4%)	<0.0001	20–30 = 43 (29.5%)	<0.0001
			F = 49 (33.6%)		31–40 = 103 (70.5%)	

TG (mg/dL)	229 (15.3%)	112 (48.9%)	M = 88 (78.6%)	<0.0001	20–30 = 29 (25.9%)	<0.0001
			F = 24 (21.4%)		31–40 = 83 (74.1%)	

HDL-C (mg/dL)	847 (56.5%)	145 (17.1%)	M = 100 (69.0%)	<0.0001	20–30 = 51(35.2%)	<0.0001
			F = 45 (31.0%)		31–40 = 94 (64.8%)	

WC (cm)	712 (47.5%)	216 (30.3%)	M = 135 (62.5%)	<0.0001	20–30 = 75 (34.7%)	<0.0001
			F = 81 (37.5%)		31–40 = 141 (65.3%)	

Premature CAD	155 (10.3%)	32 (20.6%)	M = 21 (65.6%)	0.024	20–30 = 7 (21.9%)	<0.0001
			F = 11 (34.4%)		31–40 = 25 (78.1%)	

MS: metabolic syndrome; DM: diabetes mellitus; SBP: systolic blood pressure; DBP: diastolic blood pressure; TG: triglyceride, HDL-C: high-density lipoprotein cholesterol; WC: waist circumference; CAD: coronary artery disease.

**Table 6:** Multiple logistic regression analysis using metabolic syndrome as dependent variable.

Variables	*B*	Wald	Sig.	Adjusted odds ratio	95% CI for EXP(B)
Male gender	0.6853	18.9230	0.0000	1.9844	1.4572–2.7022
Age	0.0809	35.7508	0.0000	1.0842	1.0559–1.1134
Lp. (a)	−0.0017	0.3256	0.5683	0.9983	0.9924–1.0042
Premature CAD	0.0679	0.0901	0.7640	1.0703	0.6869–1.6677
Addiction	−0.0172	0.0047	0.9452	0.9830	0.6024–1.6040
BMI	0.1145	49.9796	0.0000	1.1213	1.0863–1.1575
Constant	−7.2995	169.9954	0.0000	0.0007	

CI: confidence interval; CAD: coronary artery disease; BMI: body mass index.
